# Regulation of the Notch-ATM-abl axis by geranylgeranyl diphosphate synthase inhibition

**DOI:** 10.1038/s41419-019-1973-7

**Published:** 2019-09-30

**Authors:** Sherry S. Agabiti, Jin Li, Willie Dong, Michael M. Poe, Andrew J. Wiemer

**Affiliations:** 1 Department of Pharmaceutical Sciences, University of Connecticut, Storrs, CT 06269 USA; 2 Institute for Systems Genomics, University of Connecticut, Storrs, CT USA

**Keywords:** Lipids, Leukaemia, Apoptosis, Target validation

## Abstract

Notch proteins drive oncogenesis of many cancers, most prominently T-cell acute lymphoblastic leukemia (T-ALL). Because geranylgeranylated Rab proteins regulate Notch processing, we hypothesized that inhibition of geranylgeranyl diphosphate synthase (GGDPS) would impair Notch processing and reduce viability of T-ALL cells that express Notch. Here, we show that GGDPS inhibition reduces Notch1 expression and impairs the proliferation of T-ALL cells. GGDPS inhibition also reduces Rab7 membrane association and depletes Notch1 mRNA. GGDPS inhibition increases phosphorylation of histone H2A.X, and inhibitors of ataxia telangiectasia-mutated kinase (ATM) mitigate GGDPS inhibitor-induced apoptosis. GGDPS inhibition also influences c-abl activity downstream of caspases, and inhibitors of these enzymes prevent GGDPS inhibitor-induced apoptosis. Surprisingly, induction of apoptosis by GGDPS inhibition is reduced by co-treatment with γ-secretase inhibitors. While inhibitors of γ-secretase deplete one specific form of the Notch1 intracellular domain (NICD), they also increase Notch1 mRNA expression and increase alternate forms of Notch1 protein expression in cells treated with a GGDPS inhibitor. Furthermore, inhibitors of γ-secretase and ATM increase Notch1 mRNA stability independent of GGDPS inhibition. These results provide a model by which T-ALL cells use Notch1 to avoid DNA-damage-induced apoptosis, and can be overcome by inhibition of GGDPS through effects on Notch1 expression and its subsequent response.

## Introduction

T-cell acute lymphoblastic leukemia (T-ALL) is a rare but aggressive T-cell malignancy. T-ALL comprises ~15% of childhood and 25% of adult ALL cases^1^. While the 5-year survival rate in children (80%) has improved significantly due to advances in chemotherapy, survival in primary resistant and relapsed cases is still low^2^, and many current therapies often cause toxicity in healthy cells.

One common mutation in T-ALL is *NOTCH1*, which is altered in over 60% of T-ALL cases^3^. Notch is also mutated in other types of cancer, such as breast, colon, lung, and prostate cancer^4^. However, its role in cancer is context dependent as Notch is oncogenic in some models but functions as a tumor suppressor in others^5^. Notch signaling is evolutionarily conserved and is required at multiple points in the developmental process, particularly in T-cell development^6,7^. Notch is first processed in the Golgi by furin convertases^4^. It is then transported to the plasma membrane where it encounters its ligand (delta/serrate/LAG-2)^8^. Ligand binding promotes two cleavage events, one by Adam10 and the other by γ-secretase^9^. It has been proposed that Notch activation cannot occur without γ-secretase processing. Hydrolysis by γ-secretase processing releases the active form of Notch, the Notch internal domain (NICD), which can translocate to the nucleus, bind to transcription factors, and promote proliferation and differentiation^10^.

Recent findings suggest a role for small GTPases in Notch1 processing. Court et al. showed a requirement of Rab7 and Rab8 in Notch1 signaling^11^, while Doi et al. found that Rap1 is essential in maturation of Adam10^12^. At the same time, we identified selective inhibitors of the enzyme geranylgeranyl diphosphate synthase (*GGPS1*, or GGDPS), which can be used to disrupt the function of geranylgeranylated small GTPases^13–19^. GGDPS is an enzyme in the isoprenoid biosynthesis pathway that produces geranylgeranyl diphosphate (GGPP) whose lipid moiety is post-translationally attached to small GTPases such as Rab and Rap to promote membrane association^20,21^. Thus, we hypothesized that disrupting small GTPase lipidation with a GGDPS inhibitor would affect Notch1 signaling and display beneficial growth inhibition in T-ALL.

Because drugs such as statins and nitrogenous bisphosphonates deplete GGPP and are implicated as cancer therapeutics, it is important to understand the signaling pathways affected by GGPP depletion. Throughout these studies, we use an inhibitor of GGDPS, digeranyl bisphosphonate (DGBP), to directly deplete levels of GGPP. DGBP has a GGDPS IC_50_ of 200 nM and is well characterized, making it useful to determine how GGPP depletion leads to apoptosis^13,14^. Better understanding of the mechanisms linking GGDPS inhibition to proliferation will be useful as further developments are made regarding the preclinical development of GGDPS inhibitors^22–24^ and geranylgeranyl transferase I^25,26^ and II^27,28^ inhibitors. Here, we describe the mechanism by which GGPP depletion alters Notch1 expression and function and induces apoptosis.

## Materials and methods

### Supplies and materials

The cell lines Jurkat, Daudi, and HL-60 were obtained from ATCC (Manassas, VA, USA). Molt-4 cells were obtained from ATCC. K562 cells were obtained from Sigma Aldrich (Saint Louis, MO). RPMI-8226 cells were obtained from Coriell Institute (Camden, NJ). U2OS cells were obtained from ATCC. Cells were cultured in media containing RPMI-1640, 10% FBS, and Pen/Strep. Loucy cells were obtained from ATCC and cultured in T-cell media (RPMI-1640, 10% FBS, penicillin/streptomycin, nonessential amino acids, sodium pyruvate, 0.0004% 2-mercaptoethanol, and 10 mM HEPES, pH 7.5). Cells were grown for at least 2 weeks after thawing before experimental use, and replaced with a fresh early-passage stock after no more than 3 months. GGPP, DAPT, and coelenterazine were obtained from Cayman Chemical (Ann Arbor, MI, USA). Z-VAD-FMK was obtained from ApexBio (Houston, TX, USA). Imatinib, nilotinib, and Ku55933 were obtained from LC laboratories (Woburn, MA, USA). FITC-conjugated annexin V was obtained from Biolegend, and propidium iodide was obtained from Fisher.

Retinoblastoma protein (C-2) and c-myc (C-33) antibodies came from Santa Cruz (Santa Cruz, CA, USA). Cleaved caspase 9 (Asp330) (D2D4), p-H2A.X (S139) (20E3), ATM (D2E2), cleaved Notch1 (V1744) (D3B8) (corresponding to human V1754), and Rab7 (D95F2) antibodies were from Cell Signaling Technologies (Danvers, MA). β-actin (Poly 6221) and c-abl (8E9) antibodies were obtained from Biolegend (San Diego, CA, USA). Vinculin (hVIN-1) was purchased from Sigma. The antibody for the Notch1 internal domain (bTAN20) was purchased from the Developmental Studies Hybridoma Bank (deposited by Artavanis-Tsakonas^29^). The antibody for actin (JLA20) was obtained from the Developmental Studies Hybridoma Bank (deposited by Lin J.J.^30^).

pCIneoRL-Notch1 3′UTR was purchased from Addgene (plasmid # 84595) (deposited by Yanan Yang^31^).

### Apoptosis analysis

Cells were seeded at 100,000 cells/mL and incubated for 72 h. Cells were transferred to microcentrifuge tubes and centrifuged at 600*g* for 3 min. After the supernatant was aspirated, cells were resuspended in 200 µL of binding buffer (10 mM HEPES, 150 mM NaCl, 1 mM MgCl_2_, 5 mM KCl, and 1.8 mM CaCl_2_, pH 7.4) and transferred to polystyrene test tubes. Two microliters of PI solution were used for each condition, while three microliters of annexin V were used for each condition. Cells were mixed by vortexing, and data were acquired by using a BD Fortessa and analyzed by FlowJo.

### Cell viability

Jurkat, Molt-4, and Loucy cells in log growth were seeded at 100,000 cells/mL in 96-well plates in 100 μL and incubated for 72 h in the presence of compounds and fresh media. Ten microliters of CellQuantiBlue reagent was added per well for 2 h and scanned on a Victor5 Perkin Elmer (Waltham, MA, USA) plate reader (ex550/em600).

### Real-time RT polymerase chain reaction (RT-PCR)

Molt-4 cells were seeded at 100,000 cells/mL in 5 mL. Cells were incubated with appropriate compounds for 72 h. Total RNA was isolated with the TRIZOL (Invitrogen) according to the manufacturer’s protocol. The forward primer for NOTCH1 was 5′-AAT GCC TGC CTC ACC AA-3′. The reverse primer for NOTCH1 was 5′-CCA CAC TCG TTG ACA TCC T-3′. The forward primer for 18S was: 5′-TAA GTC CCT GCC CTT TGT AAC ACA-3′. The 18S reverse primer was 5′-GAT CCG AGG GCC TCA CTA AC-3′. RNA levels were determined with Nanodrop, and cDNA was made by using MMLV reverse transcriptase according to the manufacturer’s protocol. SYBR green (Thermo Fisher) was used according to the manufacturer’s protocol on a 7500 Applied Biosystems PCR machine. Relative mRNA levels were determined by 2^–ΔΔCt^ values.

### Luciferase assay

Cells were electroporated using established settings^19^ with 20 µg of pCIneoRL-Notch1 3′UTR DNA and 1 × 10^7^ Jurkat cells. Jurkat cells were seeded at 1 × 10^5^ in 24-well plates and treated with compounds. After 72 h, cells were centrifuged at 600 × *g* for 3 min and washed with PBS. Cells were lysed with Renilla lysis buffer (0.5× PBS, 0.025% NP-40, 1% EDTA (w/v), and freshly added 5 μM coelenterazine) and sonicated in a water bath, and immediately read on a Victor5 Perkin Elmer (Waltham, MA, USA) plate reader for counts per second. A BCA assay was carried out to determine total protein concentration (Pierce, Waltham, MA, USA).

### Western blotting analysis

Briefly, cells were resuspended in media at 250,000 cells/mL for 72 h with test compounds or solvent controls. Cells were then washed with PBS and lysed in either Whole Cell Lysis buffer (50 mM Tris, pH 8.0, 2% SDS, and 150 mM NaCl) followed by heating at 95 °C and passage through a 27½ gauge syringe or RIPA buffer (25 mM Tris-HCl, pH 7.6, 150 mM NaCl, 1% NP-40, 1% sodium deoxycholate, and 0.1% SDS) containing freshly added protease and phosphatase inhibitors including aprotinin (1 μg/mL), leupeptin (1 μg/mL), pepstatin (1 μg/mL), PMSF (200 μM), sodium vanadate (200 μM), sodium diphosphate (10 μM), sodium fluoride (50 μM), and glycerophosphoric acid (10 μM) followed by incubation for 10 min on ice and centrifugation for 10 min at 4 °C at 14,000×*g*. Lysates were quantified by BCA assay. 1× SDS was added to the sample, and the sample was then heated for 4 min at 95 °C with vortexing. Equivalent masses were loaded onto 7.5 or 12% bis-acrylamide gels depending on protein size. Proteins were transferred to nitrocellulose membranes, blocked with 5% BSA in TBS-T, and blotted with primary antibodies in blocking buffer. Alexa-Fluor 680 goat–anti-mouse IgG and IRDye 800CW goat–anti-rabbit IgG were used as secondary antibodies in TBS-T.

For the Triton X-114 separations of Rab7, membrane and cytosolic fractions were purified as described previously with some modifications^19^. Briefly, cells were resuspended in media at 250,000 cells/mL and treated with DGBP and/or test compounds for 72 h. After PBS wash, cells were lysed in Triton X-114 lysis buffer and processed for Western blots. Gels were loaded based on equivalent cell numbers/volumes.

### Statistical analysis

One-way or two-way ANOVA was used to determine statistical significance as indicated in the figure legends. In either case, follow-up tests to compare individual treatments were done by using the Tukey method in GraphPad Prism. Control conditions were compared with treatment conditions or between pairs of conditions indicated in the graphs. An α of 0.05 was used to establish significance. Bar and line graphs represent mean ± SD. Experiments were replicated at least 3 times as indicated (n = 3).

## Results

### DGBP and lovastatin, but not zoledronate or DAPT, inhibit T-ALL proliferation

**Fig. 1:**
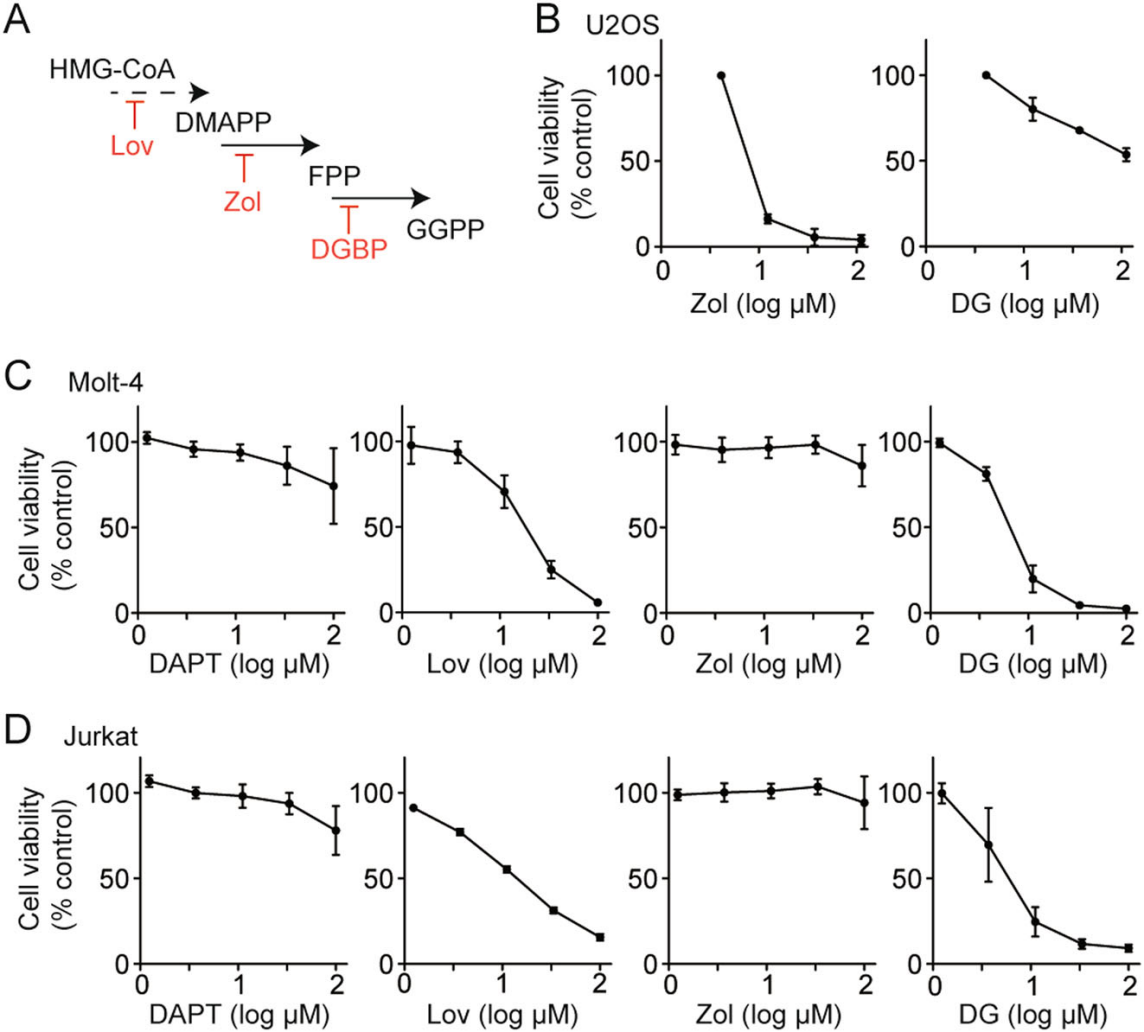
Impact of mevalonate pathway inhibitors and a γ-secretase inhibitor on cell viability. **a** Mevalonate pathway inhibitors used in this study. HMG-CoA reductase is inhibited by lovastatin (Lov), farnesyl diphosphate synthase is inhibited by zoledronate (Zol), and geranylgeranyl diphosphate synthase is inhibited by DGBP. **b** Impact of prenyl synthase inhibitors on viability of U2OS osteosarcoma cells. Graphs display means and standard deviations of two independent experiments for each compound. **c**, **d** Cell viability of Molt-4 (**c**) and Jurkat (**d**) cells after 72 h of treatment with the γ-secretase inhibitor DAPT or the mevalonate pathway inhibitors lovastatin, zoledronate, or DGBP. Graphs display means and standard deviations of three independent experiments for each compound and cell line

A recent report demonstrated that Rab GTPases regulate Notch1 processing^11^. Because Rab proteins are post-translationally geranylgeranylated, we asked whether geranylgeranylation inhibitors would affect Notch-dependent cells. We treated the T-ALL cell lines Molt-4 and Jurkat with the isoprenoid biosynthesis inhibitors lovastatin (inhibitor of HMG-CoA reductase), zoledronate (inhibitor of farnesyl diphosphate synthase), and digeranyl bisphosphonate (DGBP) (inhibitor of geranylgeranyl diphosphate synthase)^13^ (Fig. 1a) to investigate the effect on cell viability. Because zoledronate inhibits bone resorption and is active in cells of bone origin, as a control, we first assessed the impact of zoledronate and digeranyl bisphosphonate on U2OS osteosarcoma cells. Here, zoledronate was more potent than DGBP with strong activity at 10 µM, while DGBP only partially inhibited proliferation at 100 µM (Fig. 1b). In contrast, in the T-ALL cells, both DGBP and lovastatin decreased cell viability with DGBP demonstrating the stronger effect (Fig. 1c, d). Zoledronate did not impact Molt-4 and Jurkat cell viability at concentrations up to 100 µM. Thus, the pattern of zoledronate and DGBP activity is reversed in T-ALL cells in contrast to osteosarcoma. Although the active cellular concentrations are in the micromolar range, these concentrations are expected to be clinically achievable in the bone marrow environment due to the distribution kinetics driven by the bisphosphonate substructure. These values are also typical of charged bisphosphonates that require uptake by endocytic mechanisms^32^ and transporters^33^. We also evaluated the γ-secretase inhibitor DAPT, which has been proposed as a route to disrupting Notch1 processing and function. Consistent with the literature^34,35^, DAPT did not strongly impact cell viability in this time frame (Fig. 1c, d).

### GGDPS inhibition decreases expression of the Notch1 NICD and alters Rab7 localization

**Fig. 2:**
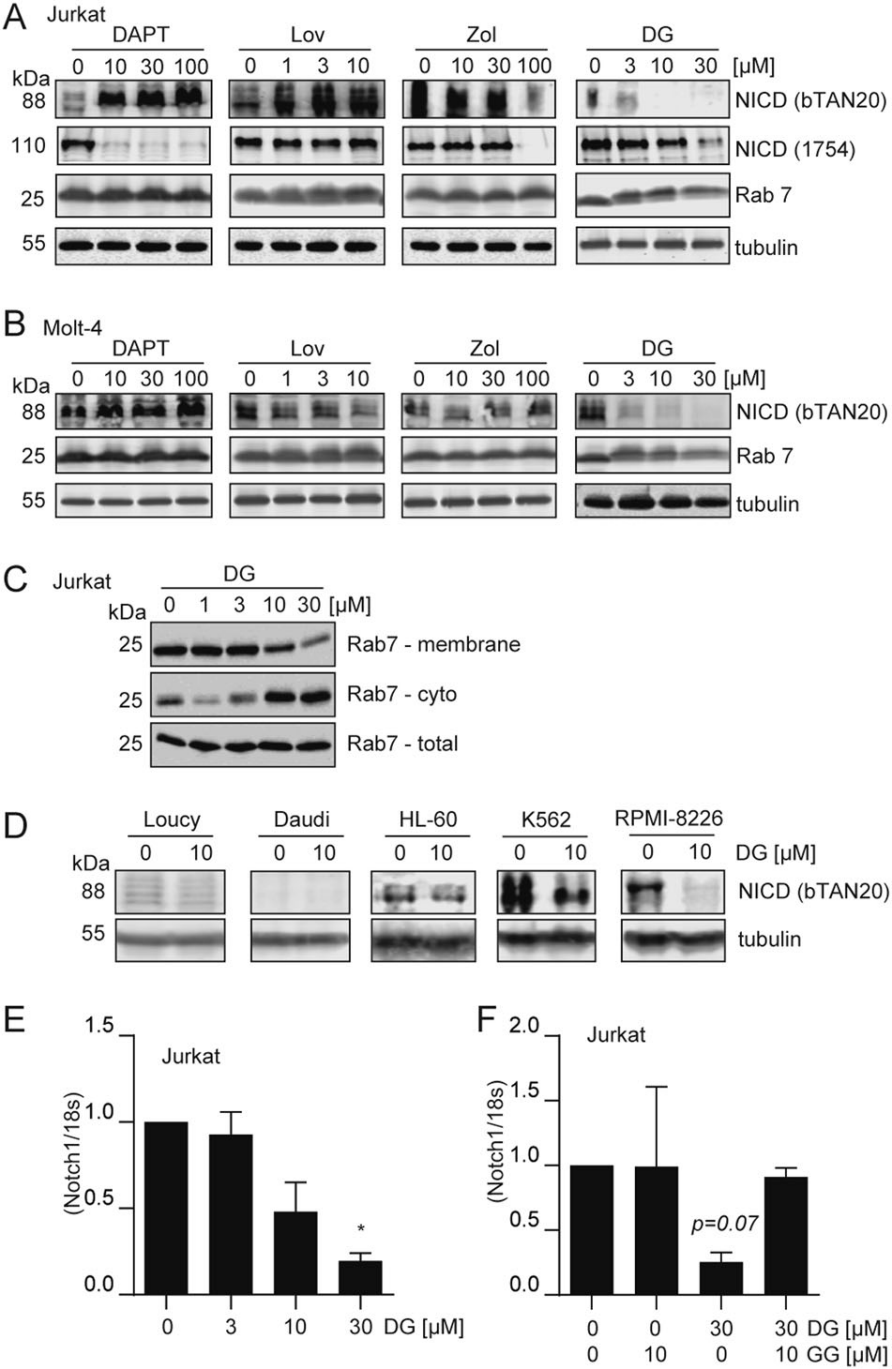
Impact of mevalonate pathway inhibitors and a γ-secretase inhibitor on Notch1 and Rab7. **a**, **b** Western blots for Notch1 intracellular domain (NICD) and Rab7 after 72 h of treatment with various concentrations of test compounds in Jurkat (**a**) and Molt-4 cells (**b**). Tubulin is shown as a loading control. Blots are representative of three independent experiments. **c** Membrane, cytosolic, and whole-cell fractions of Rab7 protein. Western blots are representative of three independent experiments. **d** Impact of DGBP on NICD expression in other hematopoietic cell lines. Western blots are representative of three independent experiments. **e**, **f** Notch1 mRNA expression relative to 18S rRNA. Cells were treated with DGBP alone or a combination with geranylgeranyl diphosphate (GGPP) for 72 h. In all graphs, the bars represent means and standard deviations of three independent experiments (*n* = 3). Statistical significance was determined by two-way ANOVA with Tukey’s post hoc analysis. *p < 0.05 versus the corresponding control

Because Notch1 is a critical oncogene that drives proliferation of T-ALL cells and it is required for proliferation of T-ALL cell lines including Jurkat cells^36,37^, we evaluated these compounds in Western blots to determine the effect on Notch1 expression. Of this panel, only DGBP completely and dose-dependently decreased Notch1 protein expression and levels of the Notch1 NICD in both Jurkat (Fig. 2a) and Molt-4 cells (Fig. 2b). The bands visualized are the predominant band on Notch1 Western blots by using the bTAN20 antibody (~88 kDa), corresponding to the predicted size of the human NICD (86 kDa)^38^. Both cell lines were resistant to γ-secretase inhibition (Fig. 1), and indeed no inhibitory effect was observed on the Notch1 NICD at concentrations up to 100 μM of DAPT in either cell line. However, use of a second Notch1 antibody specific to a cleaved form of Notch1 revealed an additional band at 110 kDa in Jurkat cells that was impacted by DAPT treatment. The 110-kDa band was not observed in the Molt-4 cells, and it was also not observed through probing with the bTAN20 antibody. Taken together, DAPT treatment increases the 88-kDa form of Notch1 and decreases the 110-kDa form of Notch1 with no impact on viability, while treatment with DGBP decreases both forms of Notch1 and impacts cell viability.

As predicted, DGBP also increased the amount of the unprocessed non-geranylgeranylated Rab7 GTPase as indicated by a slight upward shift in the apparent molecular weight (Fig. 2a, b). Because the band shift is subtle and difficult to observe/interpret, we additionally performed cellular fractionation experiments to ensure that Rab7 localization was impacted by DGBP as desired (Fig. 2c). Here, DGBP dose-dependently decreased the fraction of Rab7 associated with the membrane while increasing the fraction of Rab7 in the cytoplasm. Thus, DGBP both depletes Notch1 NICD and impacts Rab7 processing and localization. The impact of DGBP on proliferation, Notch1 NICD expression, and Rab7 processing occurs within the 10–30 µM range.

### GGDPS inhibition decreases expression of Notch1 in other hematological cell lines

In order to assess the prevalence of Notch1 regulation by GGDPS, we examined the impact of DGBP treatment on other non-T-ALL cell lines. Treatment with DGBP in various hematological cell lines revealed that DGBP reduces Notch1 NICD in cell lines expressing Notch1 (Fig. 2d). In Loucy cells, there is low Notch1 expression and no effect of DGBP on levels of NICD. However, RPMI-8226, K562, and to a lesser extent HL-60 cells expressed NICD that decreased with treatment of DGBP. Daudi cells had no expression of Notch1. Overall, in some non-T-ALL hematologic cell lines that express NICD, treatment with DGBP reduces Notch1 expression indicating that this is a conserved phenomenon, though it is most important in T-ALL cells given their dependence on Notch1.

### GGDPS controls Notch1 mRNA expression

We were surprised that inhibition of GGDPS would have such a strong effect on Notch1 expression, and asked if additional gene expression mechanisms were involved. A prior study had shown that GGDPS inhibition can also impact mRNA expression through increased flux of the reactant FPP into sterol synthesis^39^. To examine whether GGDPS inhibition could also impact Notch1 expression at the mRNA level, we tested whether or not DGBP could impact the mRNA expression of Notch1 by using real-time PCR (Fig. 2e, f). Here, treatment with DGBP dose-dependently reduced Notch1 total mRNA expression (Fig. 2e). The effect of DGBP was prevented by co-incubation with GGPP (Fig. 2f). Therefore, DGBP reduces Notch1 expression at the mRNA level through specific engagement of GGDPS. Both Notch1 mRNA reduction and alteration of Rab7 localization happen at similar concentrations and may contribute to the impact of DGBP on Notch1.

### GGDPS inhibition decreases c-myc expression

**Fig. 3:**
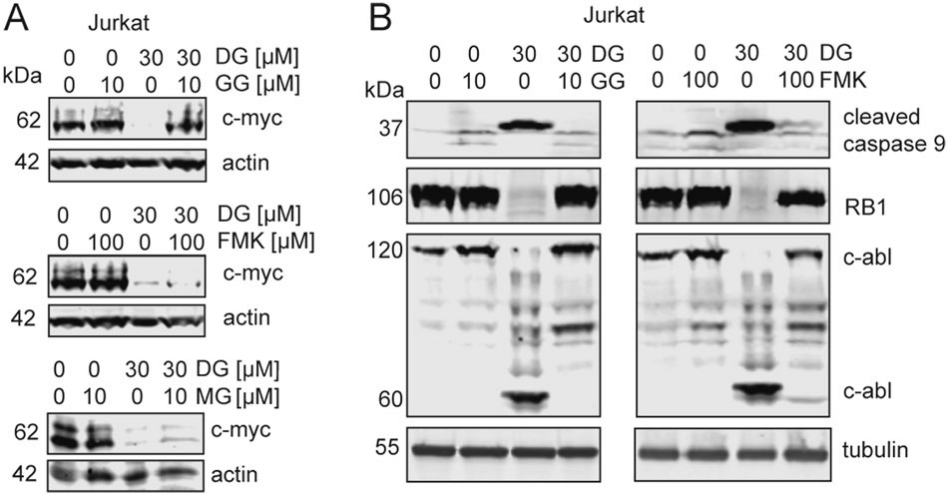
DGBP impacts c-myc expression independent of caspases and impacts RB and c-abl expression dependent upon caspases. **a** Western blots showing c-myc with DGBP treatment and co-treatment of GGPP, Z-VAD-FMK, or MG132 in Jurkat cells. Actin is shown as a loading control. All Western blots are representative of three independent experiments. **b** Western blot analysis of cleaved caspase 9, retinoblastoma (RB1), and c-abl. Tubulin is shown as a loading control. Jurkat cells were treated for 72 h with DGBP in the presence or absence of GGPP or Z-VAD-FMK. Western blots are representative of three independent experiments

We next sought to understand how reduced Notch1 expression caused by GGDPS inhibition would impact factors downstream of Notch1. Notch1 affects transcription of a variety of genes, notably including c-myc (*MYC*), which promotes oncogenesis in T-cell leukemia^10,40^. We treated Jurkat cells with DGBP and co-treated with GGPP or the caspase inhibitor Z-VAD-FMK. GGDPS inhibition decreased levels of c-myc protein expression, and co-treatment with GGPP restored the levels of c-myc (Fig. 3a). Co-treatment with Z-VAD-FMK did not rescue the effect of DGBP on c-myc (Fig. 3a), indicating that the impact on the Notch/myc axis is not downstream of caspase activation. Co-treatment of DGBP with the proteasome inhibitor, MG132, did not rescue the effect of DGBP on c-myc depletion, consistent with transcriptional regulation of c-myc downstream of Notch1 rather than increased c-myc protein degradation (Fig. 3a). Therefore, GGDPS inhibition reduces expression of Notch1 and transcription of the Notch1 effector c-myc in a way that is upstream or independent of cleaved caspases.

### GGDPS inhibition impacts signaling to caspases and apoptosis

To determine how GGDPS inhibition affects caspases, we treated Jurkat cells with DGBP and either GGPP or Z-VAD-FMK (Fig. 3b). As expected, DGBP treatment increased the expression of cleaved caspase 9. The effect of DGBP on cleaved caspase 9 was fully rescued by GGPP and Z-VAD-FMK. The pattern was also observed with retinoblastoma protein (*RB1*) expression. Furthermore, DGBP treatment decreased the 120-kDa fraction of c-abl and increased expression of the 60-kDa cleavage product of c-abl. The effect on c-abl was rescued by co-treatment with GGPP and by co-treatment with Z-VAD-FMK. Therefore, the effects of DGBP on caspase 9, RB1, and c-abl were all rescued by Z-VAD-FMK and are downstream of caspase activation. While c-abl activation is thought to be upstream of cleaved caspases in some circumstances, cleaved caspases have been previously shown at times to cleave c-abl^41^. Thus, DGBP allows for Jurkat cells to enter apoptosis through activation of caspases, depletion of retinoblastoma, and stimulation of caspase-mediated c-abl cleavage.

### c-abl mediates apoptosis induced by GGDPS inhibition downstream of caspases in T-ALL

**Fig. 4:**
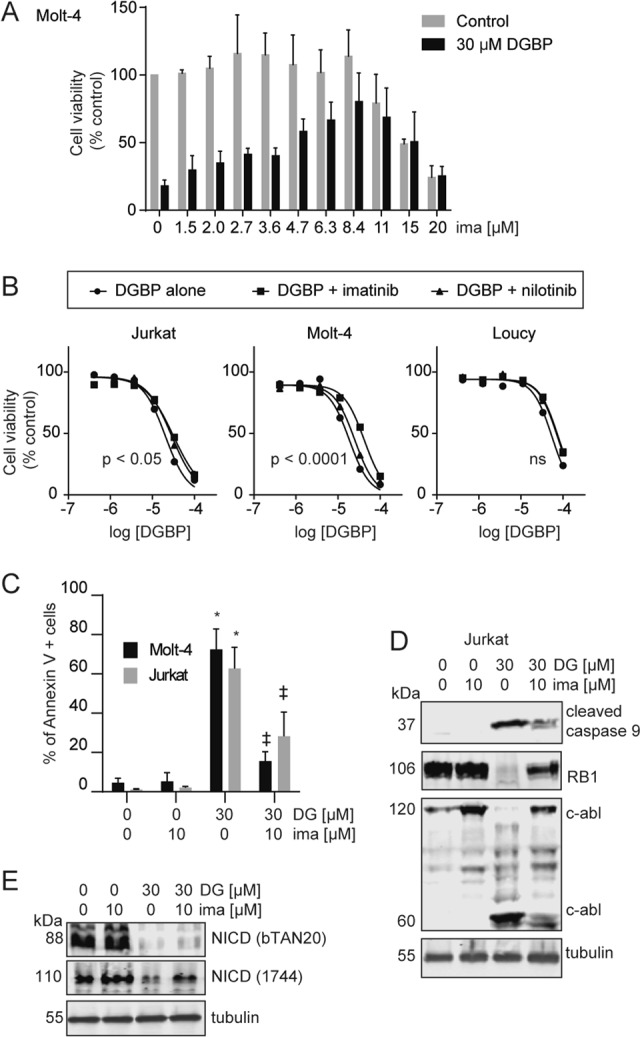
Inhibitors of c-abl are protective against apoptosis induced by DGBP, downstream of Notch1 and caspases. **a** Dose response of Molt-4 cell viability after treatment with or without 30 µM DGBP and the indicated concentrations of imatinib for 72 h. **b** Dose response of Molt-4, Jurkat, and Loucy cell viability with DGBP in combination with either 10 µM imatinib or 10 µM nilotinib for 72 h. The X axis represents the log of the molar concentration. Curves were generated by nonlinear regression by using a variable slope four-parameter model. The indicated *p* values represent confidence in whether the IC_50_ values differed between conditions. **c** Molt-4 or Jurkat cells were treated with DGBP with or without imatinib for 72 h and assessed by Annexin V staining. Flow plots shown are representative of three independent experiments. The bars represent means and standard deviations of three independent experiments (*n* = 3). The results were analyzed by using two-way ANOVA with Tukey’s post hoc analysis. *p < 0.05 versus untreated controls. ^‡^*p* < 0.05 versus DGBP-treated condition. **d** Western blotting analysis of cleaved caspase 9, retinoblastoma, and c-abl. Tubulin is shown as a loading control. Jurkat cells were treated for 72 h with DGBP in the presence or absence of imatinib. Western blots are representative of three independent experiments. **e** Western blot analysis of NICD. Tubulin is shown as a loading control. Jurkat cells were treated for 72 h with DGBP in the presence or absence of imatinib. Western blots are representative of three independent experiments

Surprised that c-abl was acting downstream of caspases in this system, we wanted to further assess the role of c-abl in apoptosis induced by GGDPS inhibition. We co-treated cells with DGBP and the c-abl inhibitors imatinib or nilotinib and determined their impact on viability, apoptosis, and protein expression. Imatinib dose-dependently reduced the impact of DGBP on Molt-4 viability with a peak impact of around 10 µM and EC_50_ of 3.5 µM (Fig. 4a). Both imatinib and nilotinib reduced the severity of DGBP on proliferation in Jurkat and Molt-4 cells but not in Loucy cells (Fig. 4b), which do not express Notch1 (Fig. 2c). We assessed the degree of apoptosis by Annexin V staining. Here, DGBP-induced apoptosis was blocked by imatinib in both Jurkat and Molt-4 cells (Fig. 4c). Co-treatment with imatinib also restored expression of the 120-kDa form of c-abl and partially reduced expression of the 60-kDa form (Fig. 4d). The effect of DGBP on cleaved caspase 9 was also partially rescued by imatinib co-treatment. Co-treatment with imatinib did not affect the ability of GGDPS inhibition to reduce expression of the 88-kDa Notch1 band (Fig. 4e). Together, c-abl acts downstream or independently of Notch1 to mediate DGBP-induced apoptosis; however, it may have functions both upstream and downstream of caspases.

### GGDPS inhibition increases the DNA-damage response

**Fig. 5:**
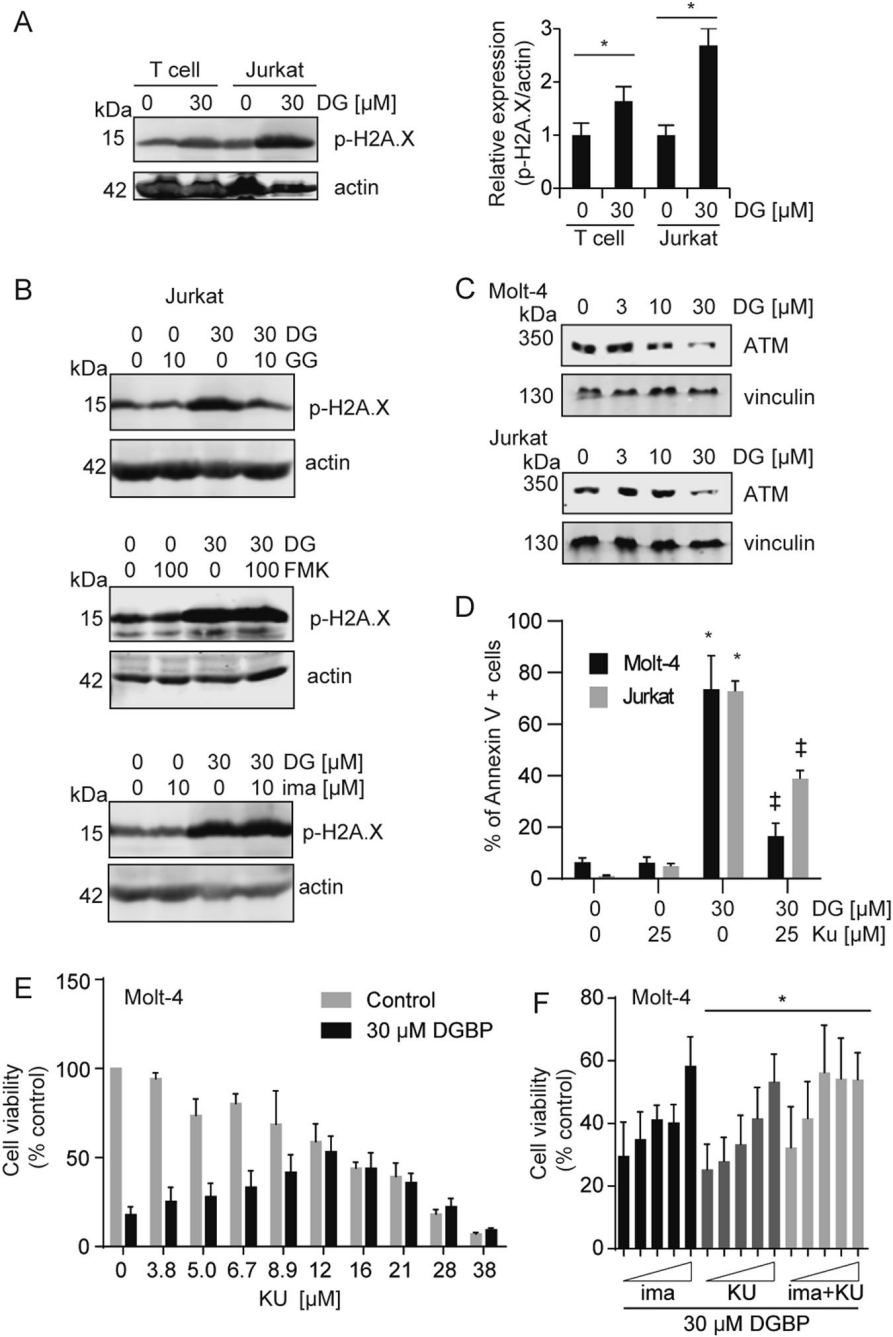
DGBP-induced apoptosis is mediated by ATM. **a** Western blot analysis of phosphorylated H2A.X in either expanded primary T cells or Jurkat cells after 72 h of treatment with DGBP, with actin shown as a loading control. **b** Western blotting analysis of p-H2A.X. Actin is shown as a loading control. Jurkat cells were treated for 72 h with DGBP in the presence or absence of GGPP, Z-VAD-FMK, or imatinib. Western blots are representative of three independent experiments. **c** Western blot analysis of ATM expression in Molt-4 or Jurkat cells treated with DGBP for 72 h, with vinculin as a loading control. **d** Annexin V staining showing 72 h of DGBP treatment along with co-treatment with or without the ATM inhibitor Ku55933 in Molt-4 and Jurkat cells. The bars represent means and standard deviations of three independent experiments (*n* = 3). **e** Dose response of Molt-4 cell viability after treatment with or without 30 µM DGBP and the indicated concentrations of Ku55933 for 72 h. The bars represent means and standard deviations of three independent experiments (*n* = 3). **f** Dose response of imatinib, Ku55933, or the combination of both inhibitors. The bars represent means and standard deviations of three independent experiments (*n* = 3). Panel (**a**) was analyzed by using a T test, while panels (**d**, **e**) were analyzed by using two-way ANOVA with Tukey’s post hoc analysis. *p < 0.05 versus controls. ^‡^*p* < 0.05 versus DGBP-treated condition

A recent report demonstrated that Notch1 can directly negatively regulate the DNA-damage response^42^. The DNA-damage response in Jurkat cells treated with DGBP is higher than that of primary T cells treated with DGBP as demonstrated by phosphorylated histone H2A.X (Fig. 5a). The effect of DGBP on p-H2A.X was rescued by GGPP, but not by Z-VAD-FMK or imatinib (Fig. 5b). Because a study by Vermezovic et al. found that Notch1 binds to the kinase regulatory domain of ataxia telangiectasia mutated (ATM), and prevents ATM activation^42^, we set out to determine if GGPP depletion regulates Notch1 to induce ATM activation and the DNA-damage response. By using an ATM inhibitor (Ku55933), we tested whether ATM was required for the apoptotic effects of GGDPS inhibition. DGBP treatment dose-dependently decreased ATM expression (Fig. 5c). Treatment of Molt-4 and Jurkat cells with Ku55933 in combination with DGBP reduced the apoptosis induced by DGBP (Fig. 5d). Ku55933 also dose-dependently reduced the impact of DGBP on Molt-4 viability with a peak impact of around 12 µM and EC_50_ of 6.8 µM (Fig. 5e). At concentrations below the EC_50_, the combination of imatinib and Ku55933 was additive (Fig. 5f).

### Notch1 depletion and cellular apoptosis induced by GGDPS inhibition are restored by γ-secretase inhibition

**Fig. 6:**
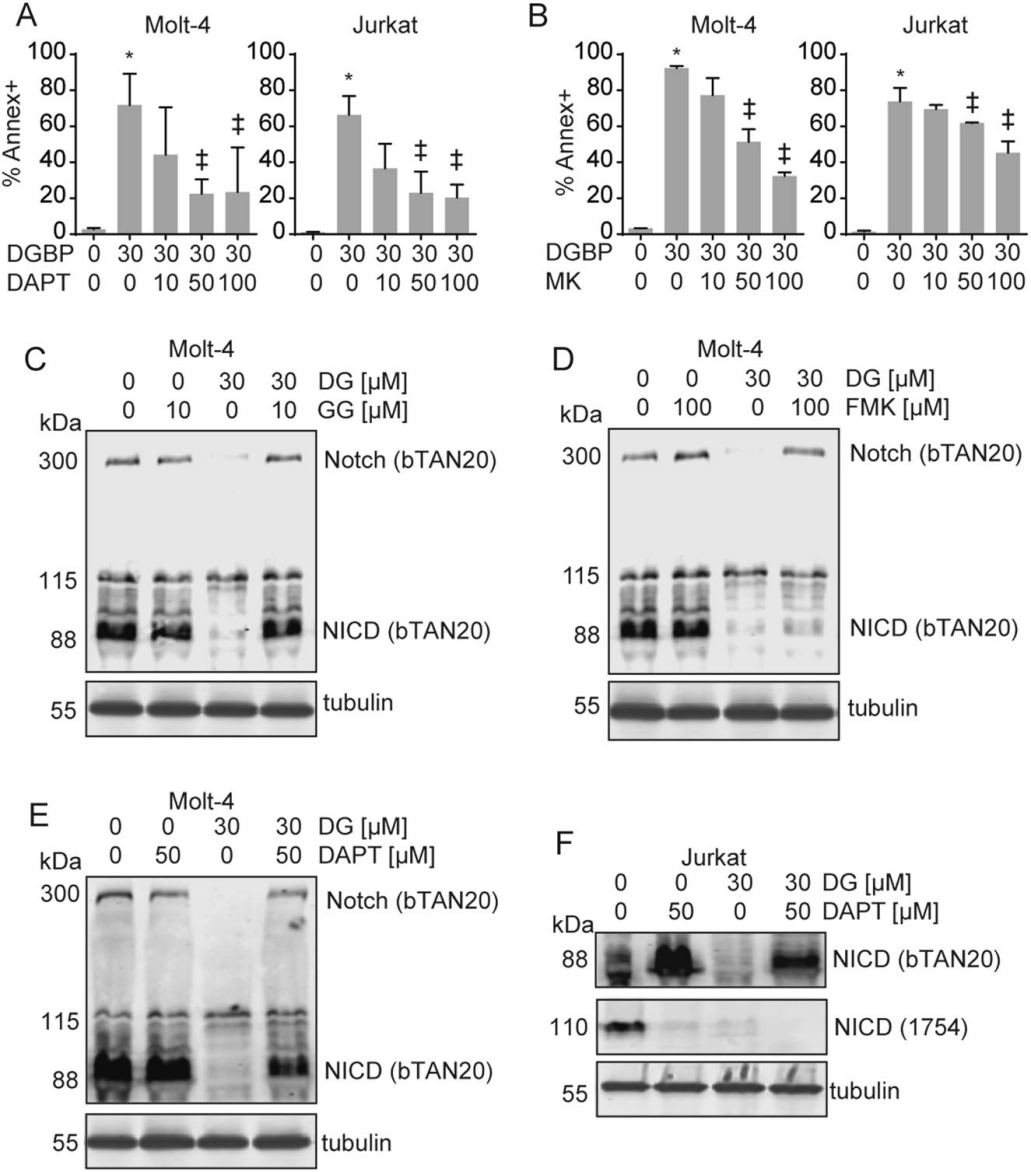
Secretase inhibitors rescue the effect of DGBP on apoptosis. **a**, **b** Annexin V staining of Molt-4 or Jurkat cells treated with DGBP and co-treated with or without (**a**) DAPT or (**b**) MK-0752 for 72 h. Graphs represent means and standard deviations of three independent experiments. Statistical significance was determined by two-way ANOVA with Tukey’s post hoc analysis. *p < 0.05 versus untreated controls. ^‡^p < 0.05 versus DGBP-treated condition. **c**, **d** Western blot of full-length Notch1 and NICD in Molt-4 cells following 72 h of treatment with DGBP and co-treatment with or without either the GGDPS product GGPP (**c**) or the caspase inhibitor Z-VAD-FMK (**d**). Tubulin is shown as a loading control. **e**, **f** Western blots of Notch1 and NICD after 72 h of treatment with DGBP and co-treatment with or without DAPT in Molt-4 (**e**) and Jurkat (**f**) cells. All Western blots are representative of three independent experiments

To further assess the relationship between GGDPS and Notch1 in T-ALL cells, we hypothesized that co-treatment with DGBP and DAPT would more strongly impact proliferation and viability than either agent alone. On the contrary, co-incubation with DGBP and DAPT actually improved cell viability relative to treatment with DGBP alone. In this regard, both DAPT and a second γ-secretase inhibitor MK-0752 dose-dependently reduced the effect of DGBP on apoptosis (Fig. 6a, b). Thus, in these T-ALL cell lines, γ-secretase works against viability and secretase inhibitors improve viability.

We next examined how this combination would affect expression of Notch1. First, as controls, we found that as expected, GGPP antagonized the reduction in Notch1 expression caused by DGBP (Fig. 6c), while co-incubation with Z-VAD-FMK did not impact the ability of DGBP to decrease NICD expression (Fig. 6d). This is further evidence that the impact of GGDPS inhibition on Notch1 expression is not a generalized nonspecific phenomenon downstream of caspase activation. To determine whether or not GGDPS reduces Notch1 expression upstream or downstream of γ-secretase cleavage, we co-treated cells with the γ-secretase inhibitor (DAPT) in combination with DGBP. DAPT restored protein levels of the 88-kDa band of Notch1 with DGBP co-incubation in both Molt-4 (Fig. 6e) and Jurkat cells (Fig. 6f). The 110-kDa Notch1 band was decreased in Jurkat cells by both DAPT and DGBP (Fig. 6f). Therefore, DGBP reduces expression of both the 88- and the 110-kDa Notch1 band and decreases cell viability, while DAPT changes the relative amounts of the 88- and the 110-kDa Notch1 band and improves cell viability.

### GGDPS controls Notch1 mRNA expression, while secretase and ATM, but not GGDPS or c-abl, control Notch1 mRNA degradation

**Fig. 7:**
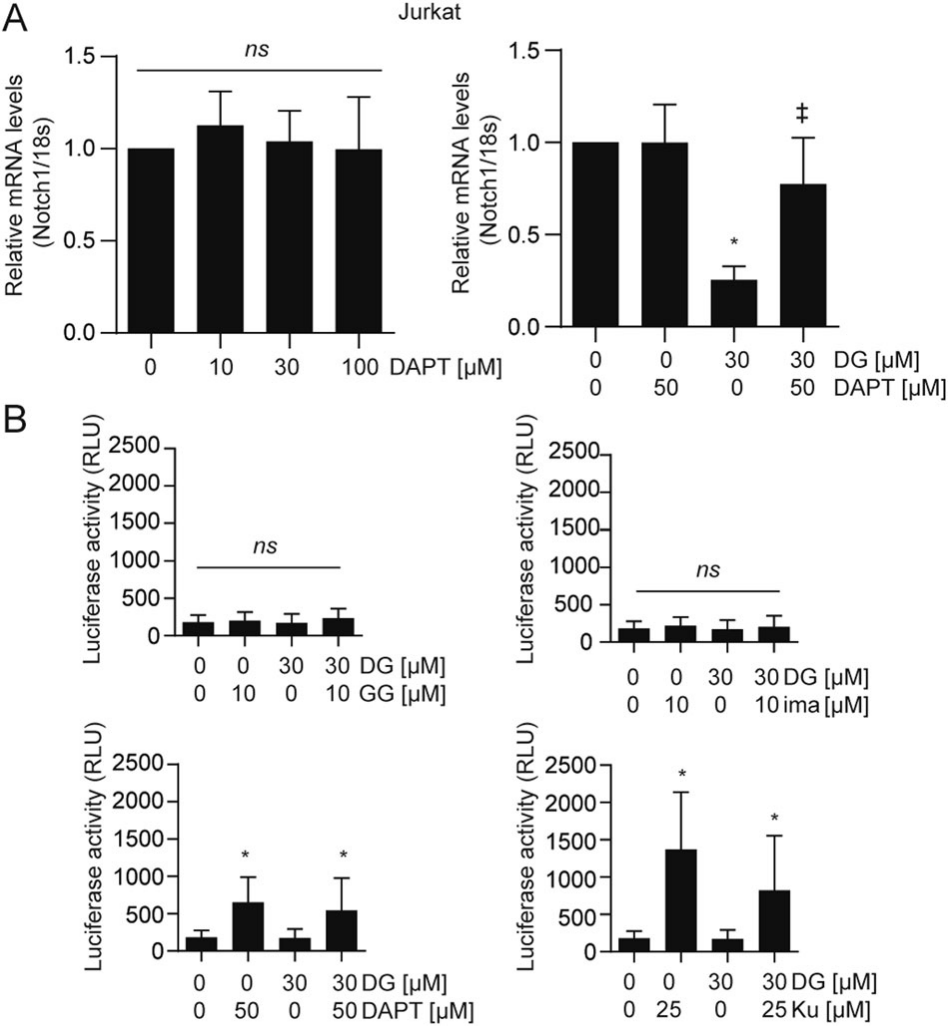
DAPT and Ku55933 restore the DGBP-induced depletion of Notch1 mRNA via decreased mRNA degradation. **a** Notch1 mRNA expression relative to 18S rRNA. Jurkat cells were treated with inhibitor alone or combinations for 72 h. **b** Luciferase activity in Jurkat cells transfected with Notch1 3′UTR. Cells were treated with inhibitor alone or co-treated with DGBP for 72 h. In all graphs, the bars represent means and standard deviations of three independent experiments (*n* = 3). Statistical significance was determined by two-way ANOVA with Tukey’s post hoc analysis. *p < 0.05 versus the corresponding control. ^‡^*p* < 0.05 versus DGBP-treated condition

Because GGDPS inhibition had affected Notch1 expression through impacts on both its processing and its mRNA expression (Fig. 2), we investigated whether γ-secretase inhibition would also impact Notch1 mRNA expression. In this regard, treatment with DAPT alone had no impact on Notch1 total mRNA expression (Fig. 7a). Surprisingly, the effect of DGBP on Notch1 mRNA expression was prevented by co-incubation with DAPT (Fig. 7a). This indicates a role for γ-secretase in regulating not only the processing of Notch1 but also its gene expression.

Notch1 mRNA expression is regulated by miR-200 interaction with the Notch1 3′UTR, which can be analyzed with a luciferase reporter construct that contains the luciferase gene flanked by the Notch1 3′UTR^31^. We assessed the ability of DGBP and the rescue agents to affect the miR-200-mediated regulation of Notch1 mRNA degradation. Treatment with DGBP did not significantly alter Notch1 mRNA stability in this system (Fig. 7b), meaning that the DGBP-induced reduction of Notch1 mRNA expression is not due to increased degradation. Treatment with imatinib also did not impact Notch1 mRNA degradation. However, treatment with DAPT and Ku55933 decreased degradation of luciferase mRNA fused to the Notch1 3′UTR leading to elevated luciferase activity. These data suggest that miR-200 not only regulates Notch, but also that miR-200 is itself regulated by secretase and ATM.

## Discussion

Notch proteins are critical to development and progression of cancers including T-ALL. In this study, we demonstrate that inhibition of the enzyme GGDPS reduces Notch1 expression and impacts factors known to be downstream of Notch1. These experiments provide evidence for regulation of Notch1 by lipid synthesis, and disruption of this mechanism can impact Notch1 expression and viability of Notch1-dependent cells.

DGBP is more effective than the secretase inhibitor DAPT and the other isoprenoid biosynthesis inhibitors at decreasing cell viability (Fig. 1) and levels of NICD (Fig. 2). The decrease in NICD is likely a result of at least two underlying mechanisms—(1) inhibition of post-translational processing of Rab proteins that are required for Notch1 trafficking, and (2) altered transcription of Notch1 itself. Immediately downstream effects include reduced c-myc expression, increased p-H2A.X, and decreased ATM expression, resulting in caspase-dependent apoptosis that is associated with decreased retinoblastoma expression and cleaved c-abl (Fig. 3). Using GGDPS inhibitors to target Notch1 signaling may be a promising therapeutic strategy because Rab GTPases are required for Notch1 processing^11^. While direct inhibitors of γ-secretase may prevent Notch1 signaling in some cases, certain mutations to Notch1 may confer insensitivity to secretase processing. Because Rab GTPases are expected to be involved in both normal and mutant Notch1 processing, disrupting GTPase function via inhibition of GGDPS allows for Notch1 disruption even in the event of mutations to Notch1.

Because γ-secretase has been proposed as an anti-Notch1 target, we evaluated combinations of secretase inhibitors and DGBP to look for potential synergies. We surprisingly found that DAPT reduces the ability of DGBP to promote apoptosis (Fig. 6), and Notch1 mRNA degradation is regulated by DAPT. The impact of DGBP and DAPT on NICD expression is more nuanced. In agreement with a prior study^43^, treatment with DAPT not only decreases expression of the NICD detected by the cleaved Notch1 antibody as expected, but also increases expression of NICD detected by a total Notch1 antibody. DGBP clearly reduces the NICD detected by both antibodies. In combination, the effect of DAPT on NICD expression can overcome the effect of DGBP, which correlates with the ability of DAPT to reduce DGBP-induced apoptosis. Therefore, GGDPS inhibition depletes NICD, while a γ-secretase inhibitor, DAPT, alters NICD expression. We speculate that this may occur because DAPT is a non-transition-state inhibitor of the γ-secretase complex that does not bind to the active site of the γ-secretase complex protease presenilin-1, but rather to an allosteric site within presenilin-1^8^. Because there are mutations along the transmembrane region of Notch1^44^, the binding conformation of the mutated α-helix may be different from the wild type. Binding of DAPT in these cases may allow for allosteric modulation rather than inhibition of the catalytic domain. Interestingly, there is overlap between the reported binding site of DAPT on presenilin-1 (aa372–467)^45^ and the binding site of Rab11 on presenilin-1 (aa374–400)^46^. Perhaps, binding of DAPT mimics binding of Rab to enable secretase processing of Notch1—though further experiments would be needed to confirm this hypothesis.

There is evidence in favor of repurposing isoprenoid biosynthesis inhibitors such as lovastatin and zoledronate for anticancer applications because zoledronate is currently used in the clinic for metastatic bone disease, and these inhibitors can lead to apoptosis^47,48^. Originally developed for non-cancer applications, these drugs have generated much interest for their potential anticancer properties. For example, a recent article showed that statins could sensitize leukemia cells to venetoclax, a BCL-2 inhibitor, in a way that is dependent upon GGPP depletion^49^. Interestingly, we observed GGDPS inhibition to affect Notch1 more strongly than HMG-CoA reductase and farnesyl diphosphate synthase inhibition^18,50^. One potential explanation for this phenomenon is that, unlike GGDPS, both HMG-CoA reductase and farnesyl diphosphate synthase are under transcriptional control through sterols^51^, which can mitigate the effects of these agents^39,52,53^. Alternatively, they may differ in rates of uptake depending on the cell line. As zoledronate has a higher charge:mass ratio than DGBP, its cellular entry is more likely to rely on endocytosis^32^.

We show that once DGBP reduces Notch1 expression, there is a twofold effect on downstream signaling: (1) decreased transcription of c-myc, and (2) induction of p-H2AX, a marker of the DNA-damage response. C-myc is a well-known downstream target of Notch1 in T-ALL^10,40,54^, while Notch1 has been reportedly involved in the DNA-damage response^42^. A potential mechanism is that DGBP-induced depletion of the NICD may free ATM to become activated and phosphorylate H2A.X, a marker for DNA damage^55^. While our studies did show rescue of DGBP-induced apoptosis by an ATM inhibitor, it remains possible that the inhibitor could disrupt function of other kinases affecting H2A.X, and further studies would be needed to conclusively determine whether ATM is mediating phosphorylation of H2A.X in this system. We propose that GGPP depletion does not directly increase DNA damage, but rather allows for cells to recognize the level of DNA damage. In this scenario, ATM would then activate cleaved caspases and initiate apoptosis^56^, while caspases would cleave retinoblastoma protein, which sits on the catalytic domain of c-abl and prevents its activation^57^. There is some precedence for Notch1 involvement in retinoblastoma protein degradation as a γ-secretase inhibitor regulates retinoblastoma to exit the cell cycle^34^. Furthermore, lovastatin and nitrogenous bisphosphonates regulate retinoblastoma protein via hypophosphorylation^58^. This reduction in retinoblastoma levels allows for caspases to cleave c-abl^41,59^. Alternatively, c-abl has been previously shown to be part of an ATM complex that activates in response to ionizing radiation^60^. However, in our studies, imatinib was not able to rescue the effect of DGBP on p-H2A.X, while the caspase inhibitor did rescue the effect of DGBP on c-abl cleavage, suggesting that the major effect of abl is downstream of caspases in this system. Together, these data indicate the connection between disruption of geranylgeranylation and induction of apoptosis in T-ALL.

Finally, we found that GGPP depletion decreases Notch1 mRNA levels but does not affect Notch1 mRNA degradation. miR-200 interacts with Notch1 signaling in cancer stem cells in breast cancer^61^ and pancreatic cancer^62^. Interestingly, only DAPT and Ku55933 impact Notch1 mRNA degradation. We predict that both of these inhibitors act on miR-200 through ATM inhibition with Ku55933 directly inhibiting ATM and DAPT acting indirectly. Further studies aimed at understanding the impact of secretase and ATM inhibitors on Notch1 expression in the context of anti-proliferative agents such as DGBP would certainly be of interest.
